# The Dynamic Mechanical Analysis of Highly Filled Rice Husk Biochar/High-Density Polyethylene Composites

**DOI:** 10.3390/polym9110628

**Published:** 2017-11-17

**Authors:** Qingfa Zhang, Hongzhen Cai, Xueyong Ren, Lingshuai Kong, Jianbiao Liu, Xuya Jiang

**Affiliations:** 1 School of Agricultural and Food Engineering, Shandong University of Technology, Zibo 255000, China; zhangqingfacll@126.com (Q.Z.); kongxiaoshuai521@163.com (L.K.); mosdut@163.com (J.L.); 18753364170@163.com (X.J.); 2 MOE Key Laboratory of Wooden Material Science and Application, College of Material Science and Technology, Beijing Forestry University, Beijing 100083, China; rxueyong@bjfu.edu.cn

**Keywords:** biochar, HDPE, composites, DMA

## Abstract

In this study, rice husk biochar/high-density polyethylene (HDPE) composites were prepared via melt mixing followed by extrusion. Effects of biochar content and testing temperature on the dynamic mechanical analysis (DMA) of the composites were studied. Morphological analysis of the rice husk biochar and composites were evaluated by scanning electron microscopy (SEM). The results showed that biochar had a positive effect on dynamic viscoelasticity, creep resistance and stress relaxation properties of the composites, but the creep resistance and stress relaxation of the composites decreased with the increase of temperature. SEM analysis showed that HDPE components were embedded in the holes of the rice husk biochar, and it is believed that strong interaction was achieved.

## 1. Introduction

High-density polyethylene (HDPE) has been widely used in agriculture, automotives, machinery, packaging and daily sundries [1] for its good heat/cold resistance, mechanical strength, chemical stability, barrier and dielectric properties [2], and is the third-most frequently used plastic used in the world [3]. Above all, HDPE has a strong plasticity and deformability [4], being widely used in the preparation of polymer composites.

Wood plastic composites (WPCs) have developed rapidly in the past several decades because of their advantages of high utilization of resources and environmental protection [5,6]. HDPE plays an important role in the development of WPC as the most commonly used matrix. WPCs are made of a mixture of biomass, thermoplastic and a certain number of additives, processed by injection molding, hot pressing and extrusion molding [7]. Over the past two decades, WPCs have made great progress due to their good properties, which include low-friction coefficient and abrasion, good plasticity, and burning resistance. There are still lots of problems, such as higher costs and poor mechanical properties because of the fibers, which have limited the scope of application [8]. In order to solve these problems, many researchers have done a lot of research into developing methods, like adding coupling agent [9], that could effectively improve the composite properties. Additionally, Li D [10] used charcoal to replace the biomass, in order to prepare composites based on ultrahigh molecular weight polyethylene (UHMWPE), the tensile strength of which is able to reach 104.7 MPa, which is far higher than WPC. Charcoal is a kind of biochar that is generally obtained from biomass pyrolysis [11], and it is completely different from biomass. There are three main products of biomass pyrolysis, including bio-oil, non-condensable gas, and biochar [12]. Bio-oil can be used in fuel boilers, engines, and turbines as fuel, and purified bio-oil could replace diesel and gasoline to a certain degree; non-condensable gas can provide heat by combustion, and can also be used as fuel; and biochar has great value and significance as a carbon sequestering agent and soil conditioner [13]. Biochar can be produced through a pyrolysis process, at under 700 °C, from a variety of agricultural and forestry wastes [14]. Common biochar includes charcoal, bamboo and straw charcoal, rice husk biochar, and so on. In recent years, biochar has been widely used in applications related to agriculture, industry, energy, the environment, and other fields. Recently, biochar has attracted much attention in the molding of composite materials. Many researchers have studied biochar as a filler for preparing biochar/plastic composites (BPCs) and proved it to be feasible, while the porous structure [15] has also provided a theoretical basis for it. This research applies the dry-blending technique and extrusion method to prepare biochar-reinforced highly filled rice husk biochar/HDPE composite, and determines its dynamic mechanical properties by using DMA.

DMA is an effective tool with the capacity to study crystalline polymers and composite materials in terms of their morphology and viscoelastic properties, including crosslinking density [16], dynamic fragility [17], dynamic/complex viscosity, storage/loss compliance, creep compliance/stress–relaxation modulus and the non-Arrhenius variation of relation times with temperature [18]. Viscoelasticity is one of the basic properties that can reflect the most prominent features of its microstructure for the polymer composite. Dynamic parameters such as storage modulus (*E*′), loss modulus (*E*″), and loss factor (tan δ) are temperature-dependent and provide information about interfacial bonding between the reinforced filler and polymer matrix of composite material [19,20]. These parameters can reflect the motion state inside the composites, and are important to the efficient use of fiber-reinforced polymer composites, which are widely used in WPC [21]. Creep is the phenomenon whereby the strain of the material increases with time under a certain temperature and constant stress, and reflects the dimensional stability of the material [22]. In particular, creep is one of the most fundamental considerations of the long-term physical properties critical to product acceptance in many engineering applications [23]. However, little information is available with respect to the effect of wood acetylation on the creep behavior of WPC [24], and research on this aspect has never been performed with regard to biochar-based composites. When a constant strain is kept at constant temperature, the stress will decrease. Stress relaxation is the result of the movement of the material under the long-term load; the faster the stress smaller, the greater the permanent deformation of the material, and the worse the carrying capacity for longer periods of time [25]. The stress relaxation is one of the most important aspects defining the viscoelastic behavior of agricultural materials [26]. The dynamic mechanical analysis has been performed on polymer matrices (thermoplastic and elastomeric) filled with both nanometer and micro-scale reinforcements, and with mechanisms of improvement including (1) any induced crystallinity, (2) restriction of the free volume of the polymeric chains due to a percolated network of nanoparticles resulting in polymeric chain stiffening, (3) interface phenomena between the reinforcement and the polymer chains, resulting synergistically in enhancement of dynamic mechanical properties, and so on [27,28,29]. The purpose of this paper is to use the extrusion method to prepare biochar-reinforced highly filled rice husk biochar/HDPE composite. The effects of biochar content on dynamical viscoelasticity, creep and stress relaxation are presented and discussed. The research in this paper is able to provide theoretical guidance for the scientific determination of application fields and provide a theoretical basis for the development of biochar-based composites.

## 2. Experiment

### 2.1. Materials

The HDPE used in this work was purchased from Qilu Petrochemical Co., Ltd. (Zibo, China). TPW604 (Tianhe, China) was used as lubricant to reduce the friction between equipment and materials. The biochar was obtained by fast pyrolysis of the rice husk powder at 500 °C using a fluidized bed reactor. All of them were sieved in order to keep them at less than 100 μm, and dried in an oven at 105 °C for 24 h prior to processing.

### 2.2. Composite Preparation

For the fabrication of composites, a high-speed mixer (JHN-15, Zhengzhou, China) was applied to blend HDPE, rice husk biochar, and the lubricant for 15 min to make them homogeneous. A twin-screw extruder (BP-8177, Dongguan Baopin International Precision Instrument Co., Ltd., Guangzhou, China) was used to prepare rice husk biochar/HDPE composite, and the barrel temperature of the extruder was controlled at 170–185 °C. The samples were prepared after water cooling from the mold. The formulation of the composite material is shown in Table 1.

### 2.3. Dynamic Mechanical Analysis

#### 2.3.1. Dynamic Viscoelasticity

The dynamic viscoelasticity of the composites was evaluated by a dynamic mechanical analyzer (Q800, TA Instruments, Baker, FL, USA). Testing samples with dimensions of 35 × 10 × 4 mm^3^ were cut from extruded rectangular bars, and were tested in a single cantilever mode with a heating rate of 5 °C/min from −50 to 150 °C at different frequencies (1, 2, 5, 10 Hz) to obtain the values for storage modulus, loss modulus and tan δ.

#### 2.3.2. The Creep Behavior

The creep tests were carried out using the Creep TTS model in the dynamic mechanical analyzer. Testing samples with dimensions of 17.5 × 10 × 4 mm^3^ were cut from extruded rectangular bars and were subjected to creep for 30 min at 25, 35, 45, 55, 65 °C, respectively, with a constant stress retention of 1 MPa. Before the creep experiment, the samples were kept at a constant temperature for 5 min to ensure uniform heat of the composites.

#### 2.3.3. The Stress Relaxation

The stress relaxation tests were carried out using the Stress Relaxation TTS model in the dynamic mechanical analyzer. The same testing samples used for the creep tests were used here, and were subjected to the same conditions, with a constant strain of 0.05%. Before the stress relaxation, the samples were kept at a constant temperature for 5 min to ensure uniform heat of the composites.

#### 2.3.4. Scanning Electron Microscopy

The rice husk biochar and composites were investigated with a field emission scanning electron microscope (FEI Sirion 200, Hongkong, China) operating at 20 kV. The powder and the fractured surface of impact section were sputtered with gold to avoid electrical charging during examination prior to processing.

## 3. Results and Discussion

### 3.1. Dynamic Viscoelasticity of the Composites

#### 3.1.1. Effect of Biochar Content on Dynamic Viscoelasticity of the Composites

**Figure 1:**
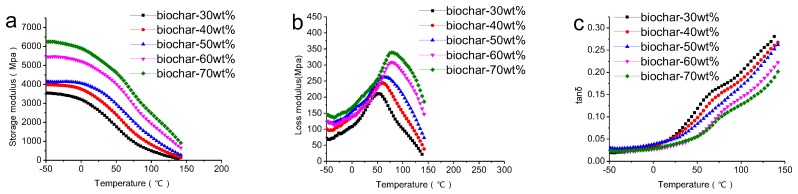
Variation of (**a**) *E*′; (**b**) *E*″ and (**c**) tan δ with temperature for corresponding composites.

The variation of *E*′, *E*″ and tan δ of different composites with temperature under 1 Hz are shown in Figure 1. It was observed that *E*′ of all the composites increased with higher biochar content across the entire temperature range due to the enhanced stiffness, as can be seen from Figure 1a, which is different from WPC [30]. The reason for this phenomenon is that, when the biochar was added as rigid filler, the HDPE component was embedded in the holes of the rice husk biochar, making them more closely connected and thus improving the deformation resistance of the composites. With the higher content of biochar, the HDPE dispersion in the composite was more homogeneous and the energy storage modulus also increased. The effect of biochar content on *E*″ of the composites is similar to *E*′; it increased with increasing addition of biochar, as can be seen from Figure 1b. This meant that the damping properties of composites improved with the increase in biochar content. Figure 1c presents the corresponding tan δ curves of the composites. It is obvious that the biochar-based composites showed a lower tan δ with the increase of biochar content, which was attributed to good interactions between the filler and the mix [31]. There were no obvious peaks on the curves of the composites up to 150 °C, which was again different from WPC [32]. As the biochar content increased, the composite became more rigid, and the restriction of the polymer chains increased. This could also explain the effect of biochar content on tan δ.

#### 3.1.2. Effect of Frequency on Viscoelastic Properties of Composites

**Figure 2:**
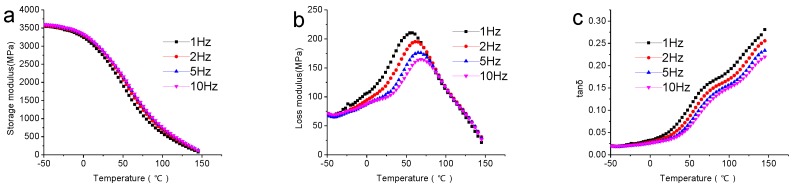
Variation of (**a**) E′; (**b**) E″ and (**c**) tan δ with frequency of composites (biochar-30 wt %).

The viscoelastic properties are mainly decided by temperature, time and frequency, which means that the effect of frequency on viscoelastic properties of composites is important. The viscoelastic properties measured over a range of frequencies (1, 2, 5 and 10 Hz) are shown in Figure 2. From Figure 2a, it was found that the storage modulus of the composites increased with the increase of frequency, but the difference was very small. The effect of frequency on *E″* of composites was obvious, in that the curve peak shifted to a higher temperature, although the peak width became more and more narrow with increasing frequency, as can be seen from Figure 2b. The tan δ of the composites decreased with the increase in frequency; the shape of the curve was not affected by frequency.

### 3.2. Creep Behavior of the Composites

#### 3.2.1. Effects of Biochar Content on Creep Behavior

**Figure 3:**
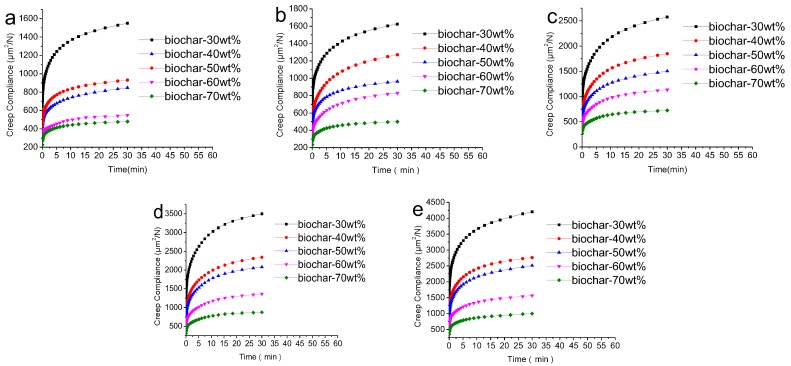
Effects of biochar content on creep behavior at different temperatures: (**a**) 25 °C; (**b**) 35 °C; (**c**) 45 °C; (**d**) 55 °C and (**e**) 65 °C.

Figure 3 shows the effects of biochar content on creep behavior at different temperatures. Three stages in the creep range of 30 min were found. The first stage is the transient creep stage, at which the creep strain represents the elastic deformation of the composites. The second stage is from fast to slow, which represents the viscoelastic deformation of the composites. The third stage is the approximately straight line of the creep curve, which belongs to the viscous deformation of the composites. From Figure 3, it was found that the shapes of the five graph curves were similar, suggesting that the creep compliance decreased with the increase of the content of biochar at the same temperature. Biochar plays an increasing role in matrix composites like fibers [33], but the effect of biochar content on creep is different. The reinforcing action of the biochar reduced the creep of the matrix with an increase in biochar content, which is consistent with fiber-reinforced composites [34].

#### 3.2.2. Effects of Temperature on Creep Behavior

**Figure 4:**
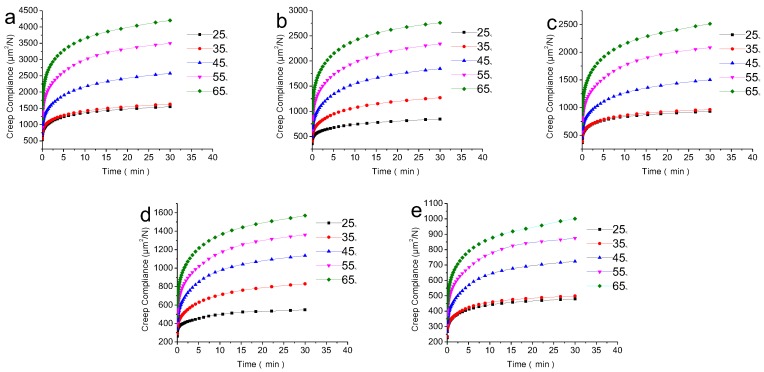
Effects of temperature on creep behavior of different composites: (**a**) biochar-30 wt %; (**b**) biochar-40 wt %; (**c**) biochar-50 wt %; (**d**) biochar-60 wt % and (**e**) biochar-70 wt %.

The creep compliance curves of the composites at different temperatures are shown in Figure 4. Creep compliance is not only related to time, but is also affected by temperature. It is clearly shown that the creep compliance of different materials increased with the increase of temperature. The reason for this was that, with the increase in temperature, the thermal kinetic energy and free volume of the matrix increased, resulting in the shortening of the relaxation time for each moving element, and a consequent increase in the creep of the matrix. The higher the temperature, the closer it is to the glass transition temperature, and the faster the creep compliance increases. This analysis could also explain the effects of temperature on creep behavior.

### 3.3. The Stress Relaxation of the Composites

#### 3.3.1. Effects of Biochar Content on Stress Relaxation

**Figure 5:**
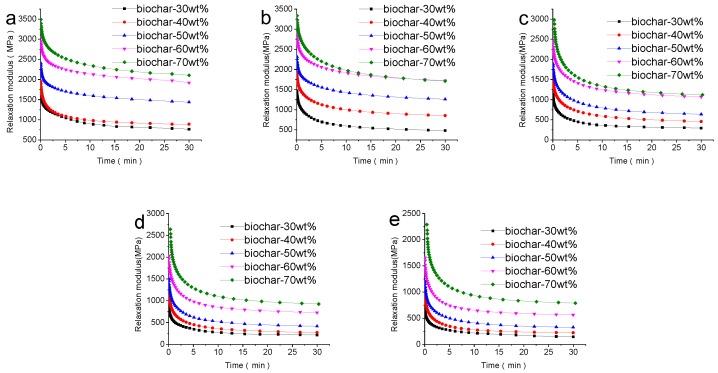
The stress relaxation modulus curves of WPC with different biochar-plastic ratio under different temperatures: (**a**) 25 °C; (**b**) 35 °C; (**c**) 45 °C; (**d**) 55 °C and (**e**) 65 °C.

The stress relaxation modulus curves of the composites with different weight ratios of biochar to HDPE at 5 temperatures are shown in Figure 5. The amount and rate of relaxation reflects the combination of the materials, and the more stress drops, the poorer the stress relaxation property of the material. As time increased, the stress relaxation modulus decreased rapidly, before gradually leveling off, indicating that the stress relaxation limit had been reached. As is shown in Figure 5, the stress relaxation modulus of composites with different biochar contents was quite varied; the stress relaxation modulus increased, and the anti-stress relaxation ability also improved with increasing biochar content, because the HDPE was more evenly dispersed in the pores of the biochar; thus, the interface was more compact, and the structure was more stable with the increase in biochar. When the filler was about 40 wt %, the stress relaxation modulus began to decrease for the WPC [35].

#### 3.3.2. Effects of Temperature on Stress Relaxation

**Figure 6:**
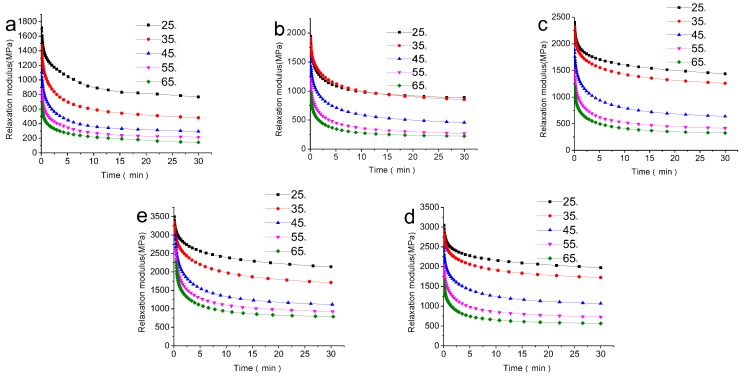
Effects of temperature on stress relaxation of different composites: (**a**) biochar-30 wt %; (**b**) biochar-40 wt %; (**c**) biochar-50 wt %; (**d**) biochar-60 wt % and (**e**) biochar-70 wt %.

Figure 6 shows the stress relaxation modulus versus time data for five different contents (30, 40, 50, 60 and 70 wt %) at different temperatures. The trends of the different curves are similar to each other, which conforms to the general law. The stress relaxation curves seem to be composed of two generally distinct parts; the stress declines sharply in the first part, which is short, but decreased gradually and lasted for a longer time in the second part [36]. What’s more, the stress relaxation modulus of the composites decreases with the increase in temperature, as the degree of freedom between the molecules in the material and then the stress response was reduced. Additionally, the stress relaxation speed of the composite increased with the increase of temperature, which was attributed to the acceleration of the internal molecular motion of the composites. The stress relaxation accelerated due to the increase of temperature [37].

### 3.4. Morphology of Biochar and the Composites

**Figure 7:**
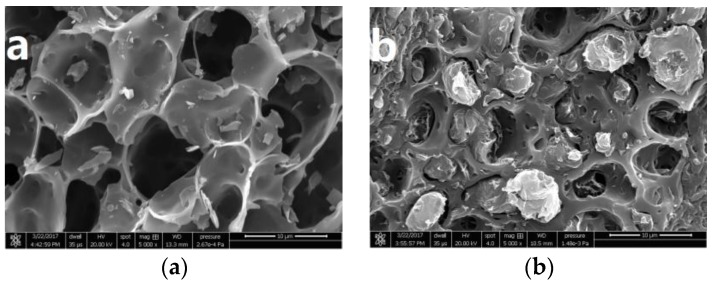
SEM images of (**a**) biochar and (**b**) the broken impact sections of composites.

Figure 7a presents the typical morphology of the rice husk biochar used in this study at a magnification of 5000×. The rice husk biochar shows an obviously porous structure, with varied pore size and shape. The carbon skeleton, pore structure and mineral matter can be observed within the macropores of these samples, which is similar to the microstructure of carbonized wood [38,39]. The large number of holes lengthwise and crosswise in the rice husk biochar might be able to strengthen the interface with the polymer matrix, due to the ultrahigh surface area, providing the possibility of the preparation of biochar-based material. Figure 7b shows the impact fracture surfaces of rice husk biochar/HDPE composite. Of particular note is that the HDPE component was embedded into the holes of the rice husk biochar, such that it is able to combine together tightly with HDPE. This structure, which is completely different from WPC [40], could transfer the stress from HDPE to the rice husk biochar, thus improving the mechanical properties of composites [41].

## 4. Conclusions

In this study, a new composite was prepared with rice husk biochar and HDPE using melt mixing following by the extrusion molding. Its dynamical mechanical properties were tested using a dynamic mechanical analyzer. The effect of the rice husk biochar/HDPE ratio on the dynamical mechanical properties of composite were studied extensively. The DMA results showed that, as a rigid filler, biochar could improve the storage modulus and decrease the loss modulus of the composites. The creep resistance and stress relaxation ability of the material increased with increasing biochar content in the composites. However, the testing temperature had a contrary effect to biochar content.

Furthermore, the SEM shows that the rice husk biochar was embedded in the HDPE. In summary, the biochar was suitable as a filler to fill HDPE to prepare composites that are environmentally friendly and can be prepared at low cost.

## Figures and Tables

**Table 1:** The content of different ingredients in the formula (wt %).

No.	1	2	3	4	5
HDPE	27	37	47	57	67
Rice husk biochar	70	60	50	40	30
TPW604	3	3	3	3	3
